# Exploring the Protective Function of Positivity and Regulatory Emotional Self-Efficacy in Time of Pandemic COVID-19

**DOI:** 10.3390/ijerph182413171

**Published:** 2021-12-14

**Authors:** Eriona Thartori, Concetta Pastorelli, Flavia Cirimele, Chiara Remondi, Maria Gerbino, Emanuele Basili, Ainzara Favini, Carolina Lunetti, Irene Fiasconaro, Gian Vittorio Caprara

**Affiliations:** Department of Psychology, Sapienza University of Rome, 00185 Rome, Italy; concetta.pastorelli@uniroma1.it (C.P.); flavia.cirimele@uniroma1.it (F.C.); chiara.remondi@uniroma1.it (C.R.); maria.gerbino@uniroma1.it (M.G.); emanuele.basili@uniroma1.it (E.B.); ainzara.favini@uniroma1.it (A.F.); carolina.lunetti@uniroma1.it (C.L.); irene.fiasconaro@uniroma1.it (I.F.)

**Keywords:** positivity, self-efficacy beliefs, anxiety, depression, COVID-19 pandemic

## Abstract

Despite several empirical studies on the 2019 coronavirus disease (COVID-19) pandemic that have highlighted its detrimental effect on individuals’ mental health, the identification of psychological factors that may moderate its impact on individuals’ behavior and well-being remains partly unexplored. The present study was conceived to examine the mediation role of regulatory emotional self-efficacy in the relationship between positivity and anxiety, depression, and perceived self-efficacy in complying with the containment measures to contrast the COVID-19 spread. Furthermore, the moderation role of age was tested. A sample of 1258 participants (64.2% women; M_age_ = 42.09, SD = 13.62) enrolled from the Italian general population answered an online survey aimed at investigating the role of individual differences in facing the COVID-19 pandemic. We opted for a snowball recruiting procedure to find participants. The online survey was disseminated through email invitation and using social media platforms (i.e., Facebook, Instagram). A multi-group path analysis model was performed using Mplus 8.4 to explore the hypothesized relations among variables. The following criteria were employed to evaluate the goodness of fit: χ^2^ likelihood ratio statistic, CFI and TLI > 0.95, RMSEA < 0.06 and SRMR < 0.08. The findings corroborated the protective role of both positivity and regulatory emotional self-efficacy in reducing individuals’ anxiety and depressive symptoms, as well as in fostering individuals’ capabilities in complying with the containment measures imposed by the government to reduce the risk of illness and to contain the spread of the virus COVID-19. Specifically, regulatory emotional self-efficacy beliefs partially mediated the relations between positivity and anxiety and depressive symptoms and fully mediated the effect of positivity on perceived self-efficacy beliefs in complying with the containment measures. These paths were equal across ages. The results of the present study appear relevant to implementing psychological interventions aimed to reduce the deleterious effects of the COVID-19 pandemic on mental health through the promotion of individuals’ optimistic orientation and emotion regulation.

## 1. Introduction

The coronavirus disease (COVID-19) pandemic has become a global health emergency. Italy was the first European country heavily affected by the COVID-19 pandemic, with the highest number of infections and victims, with approximately 120.628 deaths from the end of February to the end of April 2020 [1].

On 9 March 2020, the Italian Government imposed a strong lockdown on people requiring them to “stay home”, and adopted strict containment measures to contrast the spreading of the virus, namely, quarantine, social distancing, self-isolation, and movement restrictions. Schools, shops, bars, restaurants, and nonessential commercial activities were closed. People were allowed to leave home only if strictly necessary, for example, to go to the pharmacy/supermarket, or to go to work if their physical presence was essential [2]. The citizens were repeatedly told that the course of the pandemic and its exit mostly depended upon their attitudes and behaviors, changing profoundly their lifestyles and social relationships [3]. Although these containment measures were essential to contain the spread of the virus, they severely affected people’s habits and mental health. In particular, the increased social isolation and loneliness, the concerns about one’s health and that of their loved ones, the fear of contracting the virus and dying, and the uncertainty about the future have been associated with an increase in mental health problems, including anxiety [4,5,6], depression [4,5,6], post-traumatic stress disorder [4,5,6], sleep disorder [4,5,6], and substance use disorders [5,6].

A great number of online studies [7,8,9,10,11,12] have been proliferated in the Italian context intending to understand the impact of the COVID-19 pandemic on people’s mental health. For instance, in a study conducted on 5683 people (aged between 30 and 60 years old; 25% male) during the first week of lockdown imposed from the government during the COVID-19 outbreak, Di Giuseppe et al. [7] found that 37.8% and 51.1% of participants reported significant symptoms of depression and anxiety, respectively. Furthermore, 29.4% of participants showed clinically significant post-traumatic symptoms. In a study involving 18,147 participants (median age 38 years; 20.4% male), Rossi et al. [8] found that high rates of depression (17.3%), anxiety (20.8%), insomnia (7.3%), and post-traumatic (37%) symptoms were reported in the general population three weeks into the COVID-19 lockdown. Similarly, in a study carried out during the first four weeks of the COVID-19 lockdown, involving 2291 participants (M_age_ = 30.0, SD = 11.5; 25.3% male), Casagrande et al. [9] found that 32.1% of participants reported anxiety, 41.8% high distress, and 7.6% reported PTSD symptomatology from mid-March to 2 April. Similar findings emerged from a recent study of Amendola et al. [10], in a sample of 299 Italian adults aged 18–50 years (M_age_ = 31.14, SD = 7.99; 27.1% male), that revealed high levels of depressive and anxious symptoms and dysregulation of circadian rhythms after just over a month of COVID-19 lockdown. The detrimental impact of the COVID-19 outbreak and containment measures on mental health has also been observed over time. In a study conducted with 20,720 people (M_age_ = 40.1, SD = 14.3; 29% male), Fiorillo et al. [11] found that 12.4% of participants reported severe levels of depressive symptoms, and 17.6% of anxiety symptoms, and these symptoms significantly worsened from mid-April to May. In a longitudinal study conducted with 97 Italian young adults (aged between 19 and 29 years old) during the first 4 weeks of the COVID-19 lockdown, Parola et al. [12] found an increase in internalizing (i.e., anxiety/depression, withdrawal, and somatic complaints) and externalizing (i.e., aggressive and rule-breaking behaviors) problems in 1 month.

These findings have pointed to the need to promote the integration of mental health assessment, treatment, and psychological support into the COVID-19 response effort. In actual fact, the COVID-19 pandemic profoundly changed the agenda and the practices of health professionals, including that of psychiatrists [5,6,13], such as moving the scheduled outpatient visits to telehealth care [13].

However, although several studies have reported a high prevalence of mental health problems in the Italian population due to the COVID-19 outbreak, the identification of psychological factors that might moderate its impact on individuals’ behavior and well-being remains partly unexplored. Hence, the present study was conceived to examine the protective role that individual characteristics may exert to attenuate the impact of the COVID-19 pandemic either by contrasting the distress associated with the risk and fear of illness (i.e., anxiety state) and social isolation (i.e., depressive symptoms) and by fostering the proper behaviors to limit the contagion and to protect individual health.

The purpose of the present study fits within the theoretical and empirical contributions of the positive psychology movement [14,15,16], and the socio-cognitive theory of personality [17,18,19,20], which have paid great attention to personal and environmental determinants of individual adjustment and well-being, highlighting that the problematic aspects of the individual experience may be better addressed by strengthening and promoting potentials and resources. Specifically, through human agentic abilities, individuals are able to act on the environment through specific actions in order to achieve their goals. People who develop self-regulatory skills and self-belief also create a wide range of options, allowing the expansion of their leeway [17,18,19,20].

Amongst individual characteristics, in accordance with previous research pointing to positivity (for a review see [21]) and regulatory emotional self-efficacy (for a review see [22]) as major psychological assets to manage adversities [23,24] and foster optimal functioning [25,26,27,28], we have examined their protective role in reducing anxiety and depressive symptoms and promoting one’s efficacy in complying with the containment measures to contrast the outbreak of the virus. 

### 1.1. Positivity

Positivity, which refers to a dispositional tendency to view oneself, one’s own life, and the future under a positive outlook [25,29,30], has been conceptualized as a unique factor that encompasses self-esteem [31,32,33,34], life satisfaction [35], and dispositional optimism [36]. 

A growing body of research has attested the prominent role of positivity among the individuals’ assets that are conducive to optimal adjustment across different domains of functioning, like health [21,23,25,26], academic achievement [37], and job performance [38,39,40]. In this regard, empirical studies have pointed to the positive links of positivity with various indicators of individuals’ wellbeing, such as happiness [41], quality of interpersonal relationships [42], prosocial behavior [43], and physical health [23,25,44]. For instance, in a cross-cultural study with 1331 participants from Italy (age ranged from 20 to 92 years, M_age_ = 48.95, SD = 18.09) and 236 adolescents from Canada (M_age_ = 14.69, SD = 1.08), Caprara et al. [29] found that positivity was positively correlated with good physical health, high hedonic balance, high perceived quality of interpersonal relationships, and social support. In another study with 132 participants aged from 62 to 80 years (M_age_ = 68.21, SD = 3.99), M. Caprara et al. [44] found that higher positivity was associated with fewer health-related problems as people grew older. The positive impact of positivity on both physical and mental health has also been documented in studies conducted in clinical samples. A study by Caprara et al. [23] examined the relations between positivity and quality of life in a sample of 110 patients, aged 30–75 (M_age_ = 59.62, SD = 10.33; 40% male), interviewed at the time of cancer diagnosis, and then again after 1 year. Patients high in positivity reported fewer symptoms associated with negative affect, such as anxiety, and tended to preserve a better quality of life than patients low in positivity.

Additionally, other studies have documented the protective function of positivity against mental illness, and in particular against depression, in accordance with cognitive theories that posit negative views of the self, the world, and the future as the major aspects characterizing depressive syndrome [45]. Caprara et al. [30] showed that individuals with higher positivity were characterized by a low incidence of depressive symptoms. This protective role of positivity against depression was also supported across different countries, including Italy (689 participants; M_age_ = 19.21, SD = 1.40), the United States (1187 participants; M_age_ = 18.62, SD = 2.52), Spain (302 participants; M_age_ = 28.02, SD = 6.22), and Japan (281 participants; M_age_ = 19.54, SD = 1.66) [30]. In a two-wave longitudinal study with 742 students (48% male; M_age_ = 10.75, SD = 0.52 at Wave 1, and M_age_ = 13.38, SD = 0.94 at Wave 2), Zuffianò et al. [46] found that higher positivity was linked to lower internalizing and externalizing problems across late childhood and early adolescence. We refer the readers to Caprara et al. [21] for a more comprehensive review of the association of positivity with psychological and health outcomes.

All these empirical findings have led to viewing positivity as a trait-like basic evaluative disposition, namely, a basic attitude that equips people to face major challenges of the human condition, such as illness, aging, and death, by predisposing them to view life as worth living despite one’s limitations (see [23,25,26,47]). Positivity reflects the orchestration of several mental structures and processes that enable human beings to reflect upon their experience, to disclose their thoughts and feelings, and to make sense of their life [48]. In this regard, empirical studies have pointed to the strong associations of positivity with other adaptive individual characteristics, such as resilience, defined as individual ability to adapt successfully to changing and stressful environmental contingencies [49], positive affect, which reflects the extent to which one experiences a positive mood such as feelings of joy, interest, and enthusiasm [41], and self-efficacy beliefs, defined by Bandura [50] as a personal judgment of one’s capabilities to organize and execute the courses of action required to produce given attainments [22,24,27,28,39]. Based on these empirical studies, we reasoned that individuals who are inclined to evaluate themselves, their life, and the future in a positive outlook may be more inclined to display less anxiety and depression symptoms during the stressful COVID-19 emergency. Furthermore, positivity being a basic trait that pervasively affects how individuals appraise, view, and construe their own experience and then act [48], we reasoned that individuals who have a self-evaluative tendency to view oneself, one’s own life, and the future under a positive outlook may better regulate their behaviors, by adopting the containment measures in order to limit the COVID-19 spread.

Although previous studies pointed out the relevance of positivity for individuals’ optimal psychological functioning, to the best of our knowledge few studies [51,52] have examined the protective role of positivity on mental health during the COVID-19 pandemic, and none of them in the Italian population.

### 1.2. Regulatory Emotional Self-Efficacy

According to Bandura’s social cognitive theory [19], self-efficacy beliefs are one of the most relevant and pervasive self-regulatory mechanisms. These beliefs, which refer to one’s capabilities to organize and execute courses of action required to produce given attainments, are the expression of the self-system aimed at exercising control over the events through self-regulation. As it has been argued by Bandura [20,50], few psychological constructs may attest to the power of human agency than beliefs people hold about their capacity to cope efficaciously with arduous challenges and to face demanding situations. These beliefs, in fact, have proved to exert a pervasive influence over thought processes, motivation, affective state, and action across different domains of functioning. Likely they attest to humans’ unique capacities to orchestrate self-reflection, learning, forethought, and action regulation at service of worthy goals [18,19,20]. In this regard, a large body of research documents the pivotal role that these self-efficacy beliefs exert across domains of functioning to sustain humans’ performance and successful adaptation (for a review, see [20,50]).

Amongst self-efficacy beliefs, empirical studies have emphasized the impact of emotion regulation on attentional, cognitive, and motivational processes and have shown how failures in emotion regulation give rise to psychological problems, such as depression and delinquency (e.g., [53,54]). Focusing on emotions led to examining how beliefs people hold about their capacity to manage their emotions attest to their effective capacity to regulate their emotions and affect their life [55,56]. It is one thing to possess self-regulatory skills, but another to be able to integrate them under difficult circumstances [50].

Several studies [22,24] have attested that people’s beliefs about their capacity to express positive emotions and to regulate negative emotions exert a crucial influence on their successful development and social adaptation over the course of life, determining happiness [57], prosociality [58,59], self-esteem [27,60], and lower levels of depressive symptoms [24,55]. For example, in a two-wave longitudinal study with 464 older adolescents (aged from 14 to 19 years at wave 1, and 16 to 21 years at wave 2) Bandura et al. [24] tested the conceptual model in which one’s perceived efficacy in regulating negative emotions and expressing positive emotions predicts depression, delinquency, and prosocial behavior both directly and indirectly by their impact on perceived academic self-efficacy, social self-regulatory efficacy, and empathic self-efficacy. Regarding the outcome of our interest, they found that perceived self-efficacy to manage negative emotions predicted depression both directly and mediationally through academic and empathic self-efficacy. Moreover, perceived self-efficacy to express positive emotions predicted depression through its impact on academic self-efficacy. In a cross-cultural study involving young adults from Italy (N = 768; M_age_ = 18.72, SD = 0.90), United States (N = 1401; M_age_ = 18.86, SD = 1.00), and Bolivia (N = 301; M_age_ = 19.49, SD = 1.46), Caprara et al. [55] found that perceived self-efficacy in managing negative emotions and expressing positive emotions were negatively associated with aggressive behavior and anxiety/depression problems.

Based on prior studies [24,55] that have pointed to regulatory emotional self-efficacy beliefs as one of the key components of human agency in behavioral regulation, we reasoned that people who believe to be able to deal effectively with their negative emotions, such as sadness, anger, discouragement, and to express positive emotions, might be less at risk to develop anxiety and depression problems. Furthermore, according to previous studies [24] that have shown how higher levels of one’s perceived regulatory emotional self-efficacy represents a factor conducive to well-being through its impact on other domains of functioning, we reasoned that people who feel able to regulate their own emotions might feel capable to cope effectively with the arduous challenges connected to the stressful situation of the COVID-19 pandemic, such as changing their habits and lifestyle by conforming to the containment measures issued by the government to reduce and slow the spread of the virus. This is in line with previous studies that have shown that beliefs in personal efficacy to regulate positive and negative affective states have a generalized impact on judgments of personal efficacy in different domains [24].

### 1.3. The Present Research

**Figure 1:**
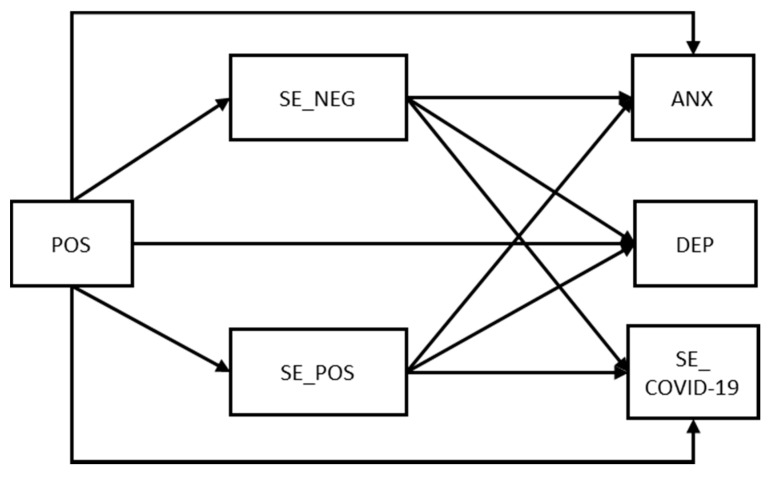
Hypothesized mediation model.

Based on the results of previous studies presented in the theoretical sections, the present study aimed to assess the protective role of positivity and regulatory emotional self-efficacy in the face of adversities carried by the COVID-19 pandemic by attenuating its negative impact on mental health (i.e., anxiety and depressive symptoms) and fostering proper behaviors (i.e., compliance with containment measures) to limit the COVID-19 spread and to protect individual health. Specifically, given that positive orientation represents a disposition that since the beginning prepares people to deal with life under a positive stance, especially in stressful conditions [21], we propose a conceptual model in which positivity contributes to individuals’ mental health both directly and indirectly through regulatory emotional self-efficacy beliefs (see Figure 1). We formulate the following hypotheses:

**Hypothesis** **1.** *Positivity negatively predicts anxiety and depressive symptoms and positively predicts one’sperceived self-efficacy in complying with the containment measures to contrast the COVID-19 spread*.

**Hypothesis** **2.** *Positivity positively predicts one’s perceived self-efficacy in regulating negative emotions and expressing positive emotions*.

**Hypothesis** **3.** *Perceived self-efficacy beliefs in regulating negative emotions and expressing positive emotions negatively predict anxiety and depressive symptoms and positively predict one’sperceived self-efficacy in complying with the containment measures to contrast the COVID-19 spread*.

**Hypothesis** **4.** *In line with the previous studies* [24,27,55] *that have highlighted the primacy of negative emotions in affecting individuals’ safety and well-being, we hypothesize that emotional self-efficacy in managing negative emotions is a better predictor of the same mental health outcomes than emotional self-efficacy in expressing positive emotions*.

**Hypothesis** **5.** *Perceived self-efficacy beliefs in regulating negative emotions (SE_NEG) and expressing positive emotions (SE_POS) mediate the relations between positivity (POS) and anxiety (ANX), depression (DEP), and perceivedself-efficacy in complying with the containment measures to contrast the COVID-19 spread (SE_COVID-19)*.

In other words, we reasoned that individuals who are inclined to evaluate themselves, their life, and the future in a positive way, might also be more prone to better regulate their emotions during the stressful situation of the COVID-19 pandemic. Emotion regulation, in turn, may reduce their anxiety and depressive symptoms, as well as foster their capability to cope with COVID-19-related demands, such as complying with the containment measures to contrast the spread of the virus. According to this hypothesis, self-efficacy beliefs may represent a mediator that significantly contributes to turning positivity into specific behaviors. To sum up, in accordance with previous distinctions between levels in the architecture of personality made by McAdams [61], we hypothesized that even if positivity and perceived regulatory emotional self-efficacy are two dimensions concerned with the self-evaluative system, they operate at different levels to predict an individual’s wellbeing. Specifically, positivity operates at a basic level by predisposing people to face the challenges of the human condition orienting their attention on resources, opportunities, and success rather than weaknesses, difficulties, and negative outcomes. While self-efficacy beliefs operate at an intermediate level by allowing people to transform their positive orientation into effective actions.

Furthermore, based on a systematic review [4] that synthesized the extant literature on the effects of COVID-19 on psychological outcomes of the general population showing that age plays a pivotal role in determining the impact of the COVID-19 outbreak and related containment measures on mental health, with older adults showing better adaptation and wellbeing than younger adults, we tested for possible age differences in the conceptual model by performing a multi-group model considering age as the grouping variable. Specifically, we computed the following three age groups: (1) from age 25 to 39 years (N = 631; M_age_ = 30.21, SD = 4.18; namely, early adulthood); (2) from age 40 to 54 years (N = 345; M_age_ = 48.09, SD = 4.23; namely, middle adulthood); and (3) over 55 years (N = 282; M_age_ = 61.36, SD = 5.90; namely, late adulthood). We hypothesized that:

**Hypothesis** **6.** *Age moderates the effects of positivity and regulatory emotional self-efficacy onanxiety, depression, and one’s perceivedself-efficacy in complying with the containment measures to contrast the COVID-19 spread*.

Finally, numerous studies [8,62,63,64] highlighted how the COVID-19 pandemic has had a more detrimental effect on females’ mental health, which were more vulnerable to developing various mental disorders during the COVID-19 pandemic, including depression and anxiety, than their male counterpart males. While the studies that have examined the impact of disadvantaged socioeconomic positions, such as lower education level, have yielded mixed results, with both lower [63,64] and higher [65,66] education levels associated with higher levels of mental disorders during the pandemic. Based on these previous empirical studies, we decided to control for the effects of gender and education level by putting them as covariates influencing all the model’s variables.

## 2. Materials and Methods

### 2.1. Procedure

The present study was part of a larger cross-sectional research project aimed at investigating the role of individual differences and the use of new technologies in facing the COVID-19 pandemic. For the purposes of the present study, we focused on examining the protective role of some individual characteristics in facing the COVID-19 pandemic. Considering the difficulty in enrolling participants due to the containment measures, an online survey was developed and disseminated using the Qualtrics software. We opted for a snowball recruiting procedure [67] in order to find participants in which research participants were asked to assist researchers in identifying other potential subjects and in disseminating the online survey. Therefore, the online survey was disseminated through email invitation to university professors, researchers, and students, which were encouraged to invite their relatives, friends, and acquaintances to participate in the study. Furthermore, in order to advertise the survey, we used social media platforms (i.e., Facebook, Instagram) where we posted a brief description of the purpose of the survey and invited people to participate in the online survey through the compilation of an anonymous link. Participants’ informed consent was obtained before starting the survey. The inclusion criteria required all participants to be at least 18 years old, to be a native Italian speaker, and to be willing to take part in the survey. The survey took approximately 15–20 min to be completed. Data collection occurred from 15 May to 22 June 2020. The study was reviewed and approved (Prot. N. 0000741 8 May 2020) by the Ethics Committee of the Department of Psychology, Sapienza University of Rome.

### 2.2. Measures

#### 2.2.1. Positivity

The positivity scale (POS) [30] was used to assess participants’ tendency to see themselves, their lives, and their future with a positive orientation. Higher scores reflect greater positivity. The scale is composed of 8 items (e.g., “I have great faith in the future”; “I am satisfied with my life”; “On the whole, I am satisfied with myself”) rated using a 5-point Likert scale ranging from 1 (strongly disagree) to 5 (strongly agree). The POS scale showed the unidimensional structure across several cultural contexts [68,69,70,71] and several empirical studies attest to its validity to predict and account for well-being and a variety of positive outcomes [26,30]. Omega (ω) reliability coefficient [72] was 0.82.

#### 2.2.2. Regulatory Emotional Self-Efficacy

Participants rated their capability to manage negative affect and express positive affect with the regulatory emotional self-efficacy (RESE) [55,56]. Three items assessed one’s capability to express positive emotions, such as joy, enthusiasm, and pride in response to success or pleasant events (e.g., “How well can you express joy when good things happen to you?”; “How well can you rejoice over your successes?”; ω = 0.89), while five items assessed one’s capability to regulate negative emotions such as anger/irritation and sadness/despondency in response to adversity or frustrating events and one’s capability to avoid being overcome by emotions such as anger, irritation, despondency and discouragement (e.g., “How well can you keep from getting discouraged in the face of difficulties?” and “How well can you avoid flying off the handle when you get angry?”; ω = 0.81). For the current study, we asked participants to respond thinking how they felt in the lockdown period, during the COVID-19 pandemic. The participants rated the strength of their self-efficacy beliefs on a 5-point Likert scale ranging from 1 (not well at all) to 5 (very well). The RESE scale has been validated and used widely in several countries [55,73] while a growing body of evidence attests that people’s beliefs about their capacity to express positive emotions and to control negative emotions exert a crucial influence on their successful development and social adaptation over the course of life [24,28,55,57,59].

#### 2.2.3. State Anxiety

A brief version of the state-trait anxiety inventory (STAI) was used to assess the state and trait components of anxiety [74]. In the present study, we focused on the state component of anxiety, which refers to how one feels at the moment. Participants rated (ranging from 1 (not at all) to 4 (very much so)) 8 items tapping their tendency to feel anxious in that moment (e.g., “I feel nervous”, “I am tense”; ω = 0.90). The STAI is the most widely used self-report questionnaire to assess anxiety; it has appeared in over 3000 studies and is available in over 30 languages [74]. Several empirical studies have been carried out to evaluate its validity, showing moderated correlations between STAI and other measures of anxiety, worry, and depression [75,76].

#### 2.2.4. Depressive Symptoms

A brief 10-item version of the Center for Epidemiologic Studies Depression Scale (CES-D) [77] was used to assess participants’ depressive symptoms experienced in the past week (e.g., “I felt depressed”; “I felt that I could not shake off the blues even with help from my family or friends”; “I was bothered by things that usually don’t bother me”), on a 4-point scale ranging from 1 (rarely or none of the time (less than 1 day)) to 4 (most or all of the time (5–7 days)). The CES-D is widely used as a screening tool to identify persons at risk for clinical depression [78,79]. The validity of this measure has been corroborated [77,80] and several empirical studies attested to its correlation with self-esteem and anxiety [81]. Omega (ω) reliability coefficient [72] was 0.90.

#### 2.2.5. Self-Efficacy in Complying with the Containment Measures

For the aims of the study, a 7-item ad hoc scale was developed to assess an individual’s capability to comply with the containment measures issued by the government to reduce and slow the spread of COVID-19. The item formulation process followed Bandura’s guidelines [82]. In particular, the items were phrased in terms of can do rather than will do, and sufficient gradations of difficulties were built. The following 7 items were developed: “How well you can include in your daily routine practices to contain the spread of COVID-19 (for example, hand washing often, wearing a face mask)”; “How well can you adjust your personal needs to the changing government requests?”; “How well you can resist the urge to leave home to visit your relatives, partners, and friends when is not necessary?”; “How well can you respect the government restrictions even if you disagree?”; “How well you can avoid social gatherings and stay at least 1 m away from other people when going outside (for example, to the park)”; “How well you can avoid social gatherings and stay at least 1 m away from other people when going to see your relatives/partners?”; and “How well you can resist the urge to not use safety devices (such as a face mask) when going outside (for example, to the supermarket, to the park)?”. The participants rated the strength of their self-efficacy beliefs on a 5-point Likert scale ranging from 1 (not well at all) to 5 (very well). To investigate the dimensionality of these sets of self-efficacy items, exploratory factor analysis was carried out on the data. We extracted factors using the principal axis factoring method. In order to determine the number of factors to retain, we examined the eigenvalues [83]. The analysis of eigenvalues suggested a one-factor solution, given that the ratio of the first to the second eigenvalues was greater than 2 (the first two eigenvalues were 3.39 and 0.82) [84]. The first factor accounted for 48.55% of the total variance. All loadings were greater than 0.40, ranging from 0.47 to 0.80. Omega (ω) reliability coefficient [72] was 0.80.

#### 2.2.6. Control Variables

Participants were asked to report their gender (0 = male, 1 = female) and level of education. As income and education are highly correlated [85], the level of education (from 1 = middle school degree to 5 = master’s degree or higher) was used as the indicator of socioeconomic status (SES).

### 2.3. Strategy of Analysis

Preliminarily, means, standard deviations, and zero-order correlations among the study variables were calculated using SPSS (Version 23, SPSS Inc., Armonk, NY: IBM Corp.). Then, path analysis modeling was performed using Mplus 8.4 [86] to explore the hypothesized relations among variables. The following criteria were employed to evaluate the goodness of fit: chi-squared likelihood ratio statistic (χ^2^), Tucker and Lewis Index (TLI), comparative fit index (CFI), and the root mean square error of approximation (RMSEA) with 90% confidence interval (CI), and the standardized root mean square residual (SRMR). χ^2^ indicates the difference between observed and expected covariance matrices. Values closer to zero indicate a better fit. Because χ^2^ is sensitive to sample size—therefore obtaining a nonsignificant chi-square becomes increasingly unlikely with complex models and large sample sizes—other measures of fit have been considered to evaluate the model fit [87]. SRMR is an absolute index of the discrepancy between reproduced and observed covariance matrices. Values of SRMR lower than 0.08 indicate a good model fit [88]. RMSEA is a measure of the discrepancy per degree of freedom for the model. Values of the RMSEA equal or lower than 0.06 indicate an acceptable model fit [89]. The CFI [90] assesses the improvement in fit going from the baseline model to the postulated model. Values equal or higher than 0.95 are considered adequate for good models [88]. Finally, the TLI [91] measures a relative reduction in misfit per degree of freedom model. Values equal or higher than 0.95 are considered adequate for good models [88].

Finally, in order to test for possible moderation by age, firstly, we divided the participants into three age groups: (1) from age 25 to 39 years (N = 631; M_age_ = 30.21, SD = 4.18; namely, early adulthood); (2) from age 40 to 54 years (N = 345; M_age_ = 48.09, SD = 4.23; namely, middle adulthood); and (3) over 55 years (N = 282; M_age_ = 61.36, SD = 5.90; namely, late adulthood). Then, we performed a multi-group path analysis model considering age (1 = early adulthood, 2 = middle adulthood, and 3 = late adulthood) as the grouping variable, which simultaneously estimated the same pattern of relations among the variables across the age groups. In this approach, equivalence among different groups is evaluated by constraints that impose identical estimates for the model’s parameters [92,93]. In the Mplus framework, the plausibility of these constraints is examined with the modification indices and the χ^2^ difference test between nested models (i.e., constrained models vs. the baseline unconstrained model) [94].

## 3. Results

### 3.1. Participants’ Characteristics

The sample of the present study consisted of 1.258, including 453 (35.4%) men, 820 (64.2%) women, and 5 other (0.4%). The age of the participants ranged between 25 to 81 (M_age_ = 42.09, SD = 13.62). Of the participants, 40.9% had a master’s degree or higher (e.g., Ph.D.), 36.8% had a high school degree, 14.7% had a bachelor’s degree, and 7.6% had a middle school degree. Additionally, participants reported that 37.5% were married, 20.2% single, 17.5% cohabiting, 16.1% in an exclusive relationship but not living together, 3.8% divorced, 2.5% separated, 2% widowed, and 0.4% other. In order to test the hypothesized model across age, they were divided into three age groups: (1) from age 25 to 39 years (N = 631; M_age_ = 30.21, SD = 4.18; namely, early adulthood); (2) from age 40 to 54 years (N = 345; M_age_ = 48.09, SD = 4.23; namely, middle adulthood); and (3) over 55 years (N = 282; M_age_ = 61.36, SD = 5.90; namely, late adulthood).

### 3.2. Preliminary Analyses

Means and standard deviations for the main study variables are presented in Table 1.

Correlations among the study variables are reported in Table 2. All variables resulted significantly correlated. Specifically, positivity and perceived self-efficacy beliefs in regulating negative emotions and expressing positive emotions are negatively correlated to anxiety and depressive symptoms and positively correlated to one’s perceived self-efficacy in complying with the containment measures to contrast the COVID-19 spread (SE-COVID-19). Furthermore, positivity is positively related to one’s capability to regulate negative emotions and express positive ones. Finally, anxiety and depression are positively correlated, while SE-COVID-19 is negatively correlated with anxiety and depression.

### 3.3. The Mediational Multi-Groups Path Analysis Model

The hypothesized constrained model, with the structural relations among variables constrained to equality across age, showed a good fit to the data: χ2(51) = 49.061, *p* = 0.551, CFI = 1.00, TLI = 1.00, RMSEA = 0.00 (0.00–0.03), SRMR = 0.05. The comparison between this model and the baseline unconstrained model (i.e., model with no equality constraints on parameter estimates across age) resulted in a non-significant chi-square difference test: Δχ^2^(30) = 35.124, *p* < 0.05, revealing that all parameters were equal across age. Overall, the posited model accounted for 35% of the variance in state anxiety, for 32% in depressive symptoms, and 9% in self-efficacy in complying with the containment measures.

In order to determine significant mediation effects, bootstrapping analysis with 99% confidence interval and 10,000 resampling paths was used. The mediators were considered to have a significant effect only when the 99% CI did not include zero [95].

#### 3.3.1. Positivity, Regulatory Emotional Self-Efficacy, Anxiety, and Depression Symptoms

**Figure 2:**
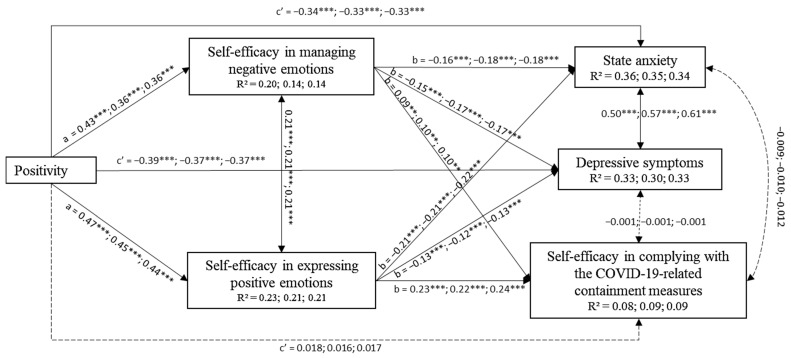
Links among positivity, regulatory emotional self-efficacy beliefs and mental health outcomes. Mediation diagram: a, b, and c’ are path coefficients representing standardized regression weights. The c’ path coefficient refers to the direct effect of positivity on mental health outcomes. Non-significant paths are represented by dashed lines. Moderation effects: standardized coefficients are reported for early adulthood, middle adulthood, and late adulthood, respectively. The comparison between the constrained model and the baseline unconstrained model (i.e., model with no equality constraints on parameter estimates across age) resulted in a non-significant chi-square difference test: Δχ^2^(30) = 35.124, *p* < 0.05, revealing that all parameters were equal across age. Finally, to ease the interpretation, the effects of control variables (i.e., gender, SES) were not reported. ** *p* < 0.01; *** *p* < 0.001.

As shown in Figure 2, in accordance with our hypothesis, positivity contributed to anxiety and depression symptoms both directly and indirectly through its effect on both perceived self-efficacy beliefs in managing negative emotions (SE_NEG) and in expressing positive emotions (SE_POS). In particular, SE_NEG and SE_POS partially mediated the contribution of positivity on state anxiety and depressive symptoms.

Specifically, regarding state anxiety, the indirect effect of positivity on state anxiety via SE_NEG was −0.068 (*p* < 0.001; 99% C.I. = −0.102; −0.041), −0.064 (*p* < 0.001; 99% C.I. = −0.096; −0.039), and −0.065 (*p* < 0.001; 99% C.I. = −0.100; −0.040), respectively, for early adulthood, middle adulthood, and late adulthood. The indirect effect of positivity on state anxiety via SE_POS was -0.101 (*p* < 0.001; 99% C.I. = −0.140; −0.069), −0.096 (*p* < 0.001; 99% C.I. = −0.133; −0.065), and −0.097 (*p* < 0.001; 99% C.I. = −0.137; −0.066), respectively, for early adulthood, middle adulthood, and late adulthood.

Regarding depressive symptoms, the indirect effect of positivity on depressive symptoms via SE_NEG was −0.060 (*p* < 0.001; 99% C.I. = −0.096; −0.026), −0.060 (*p* < 0.001; 99% C.I. = −0.093; −0.034), and −0.060 (*p* < 0.001; 99% C.I. = −0.095; −0.033), respectively, for early adulthood, middle adulthood, and late adulthood; the indirect effect of positivity on depressive symptoms via SE_POS was −0.064 (*p* < 0.001; 99% C.I. = −0.100; −0.035), -0.057(*p* < 0.001; 99% C.I. = −0.092; −0.022), and −0.056 (*p* < 0.001; 99% C.I. = −0.090; −0.023), respectively, for early adulthood, middle adulthood, and late adulthood.

These findings highlight that individuals that evaluated themselves, their life, and the future in a positive way, felt more able to regulate their emotions during the stressful situation of the COVID-19 pandemic, which, in turn, reduced their anxiety and depressive symptoms. The protective role of positivity against anxiety and depression symptoms is also confirmed by its direct effect, showing that positivity predicted lower anxiety and depressive symptoms.

Finally, SE_NEG and SE_POS, as well as state anxiety and depressive symptoms, were positively correlated.

#### 3.3.2. Positivity, Regulatory Emotional Self-Efficacy, and Perceived Self-Efficacy Beliefs in Complying with the Containment Measures

As shown in Figure 2, regulatory emotional self-efficacy completely mediated the contribution of positivity on perceived self-efficacy in complying with the containment measures to contrast the COVID-19 spread (SE_COVID-19).

Specifically, the indirect effect of positivity on SE_COVID-19 via SE_NEG was 0.039 (*p* < 0.01; 99% C.I. = 0.002; 0.076), 0.035 (*p* < 0.01; 99% C.I. = 0.002; 0.067), and 0.037 (*p* < 0.01; 99% C.I. = 0.002; 0.071), respectively, for early adulthood, middle adulthood, and late adulthood. The indirect effect of positivity on SE_COVID-19 via SE_POS was 0.111 (*p* < 0.001; 99% C.I. = 0.066; 0.151), 0.099 (*p* < 0.001; 99% C.I. = 0.057; 0.139), and 0.104 (*p* < 0.001; 99% C.I. = 0.062; 0.145), respectively, for early adulthood, middle adulthood and late adulthood.

In other words, positivity predicted the individuals’ capability to cope effectively with COVID-19 challenges and government demands, such as changing their habits and lifestyles to contain the spread of the virus, through its effect on fostering emotion regulation. This means that self-efficacy beliefs operate as individual factors that allow people to transform their positive orientation into effective actions and beliefs.

#### 3.3.3. The Effects of Gender and SES

The standardized effects of gender and SES are reported in Table 3. SES was positively related to positivity across age groups, and positively related to SE_POS only in late adulthood. Gender was negatively related to SE_NEG, and positively related to state anxiety and depressive symptoms across age, whereas it positively related to SE_POS only in early adulthood, and positively related to SE_COVID-19 only in middle adulthood.

### 3.4. Alternative Model

As a further test of the appropriateness of the hypothesized model, an alternative plausible model was tested in which the direction of causation was reversed. Specifically, in this alternative model, positivity was posited as a mediator in the relationship between regulatory emotional self-efficacy beliefs in dealing with negative emotions and in expressing positive emotions and the mental health outcomes (i.e., state anxiety, depressive symptoms, and perceived self-efficacy in complying with the containment measures). We examined this model in the multi-group path analysis modeling framework, with age as the grouping variable. The Akaike information criterion (AIC) is particularly well suited for comparing the adequacy of non-nested models [56]. The lower the AIC index, the better the goodness-of-fit. The AIC for the hypothesized model was 12047.369. Although this alternative model yielded a reasonable fit to the data, χ2 (51) = 53.419, *p* = 0.381, CFI = 0.99, TLI = 0.99, RMSEA = 0.011 (0.00–0.03), SRMR = 0.07, and AIC = 12051.728, the hypothesized model provided a lower AIC than the alternative model’s AIC, revealing that the hypothesized model was the best.

## 4. Discussion

At a distance of almost two years from the inception of the COVID-19 pandemic, several studies document its negative effects on individuals’ mental health, such as anxiety and depression (for a review see [4]). Given the prolongation of the COVID-19 pandemic, the identification of protective psychological factors that may moderate its impact on individuals’ behavior and well-being is necessary. To this aim, we investigated the protective role of both positivity and regulatory emotional self-efficacy on individuals’ mental health during the COVID-19 pandemic in Italy. Specifically, we aimed to examine the direct and indirect effect, through regulatory emotional self-efficacy beliefs, of positivity on anxiety, depressive symptoms, and perceived capability to cope with COVID-19 related demands, such as the capability to comply with the containment measures issued by the Italian government to contain the spread of the virus and reduce the risk of illness (SE_COVID-19). Possible differences among ages were also explored.

### 4.1. Direct Effects of Positivity on Mental Health Outcomes

Overall, in line with our hypothesis 1, positivity negatively predicted anxiety and depressive symptoms and positively predicted one’s self-efficacy in complying with the containment measures to contrast the COVID-19 spread. In other words, individuals who evaluated themselves, their life and the future in a positive way, reported lower anxiety and depressive symptoms and were more capable to adapt their habits and lifestyle to the containment measures adopted to reduce the spread of the virus and contain the risk of illness. Moreover, in line with our hypothesis 2, positivity positively predicted one’s perceived emotional self-efficacy in regulating negative emotions and expressing positive ones. These findings corroborate previous results pointing to the protective role of positivity against mental health [21,23,25,26], and show its strong associations with other adaptive individual characteristics, such as self-efficacy beliefs [22,24,27,28,39]. Moreover, our results supported the studies that have led to viewing positivity as a trait-like basic disposition that predisposes people to view life as worth living despite having to face the hard challenges and frailty of the human condition, and thus conducive to happiness [41].

### 4.2. Direct Effects of Regulation Emotional Self-Efficacy Beliefs on Mental Health Outcomes

In accordance with our third hypothesis, perceived regulatory emotional self-efficacy beliefs in managing negative emotions and expressing positive emotions negatively predicted anxiety and depressive symptoms and positively predicted one’s self-efficacy in complying with the containment measures to contrast the COVID-19 spread. In other words, individuals who were able to regulate their negative emotions and manage their positive ones were less at risk to develop anxiety and depressive symptoms, and more prone to feel able to adopt the containment measures in their lifestyle. These findings are concordant with previous studies [24,55] showing that perceived self-efficacy in managing negative emotions and expressing positive emotions were negatively associated with anxiety and depression problems, and positively related to other self-efficacy domains [24]. In addition, contrary to our hypothesis 4, emotional self-efficacy in expressing positive emotions contributes to individuals’ good adaptation more than regulatory emotional self-efficacy in dealing with negative emotions. In particular, while individuals’ efficacious beliefs in handling their negative emotions seem critical for reducing depressive symptoms, their efficacious beliefs about expressing positive emotions play a more relevant role in individuals’ adaptation to the stressful COVID-19 situation (i.e., state anxiety and SE_COVID-19). These findings supported the literature that has emphasized the positive effect of positive emotions in human functioning and adjustment (e.g., [96,97,98]).

### 4.3. The Mediation Effect of Regulatory Emotional Self-Efficacy on the Relation of Positivity and Mental Health Outcomes

Results of path analysis testing for indirect effects corroborate our hypothesis 5 showing that individual’s regulatory emotional self-efficacy beliefs are one mechanism by which positivity contributed to mental health. Specifically, self-efficacy beliefs in managing negative emotions and expressing positive emotions partially mediated the relations between positivity and anxiety and depressive symptoms. This means that positivity represents a protective factor able to reduce levels of individuals’ anxiety and depressive symptoms during the pandemic COVID-19 emergency not only by fostering their capacity to deal with negative affect and express positive affect, but also directly predisposing them to be less prone to anxiety and depression during the stressful situation of the COVID-19 pandemic.

Conversely, regulatory emotional self-efficacy completely mediated the relation between positivity and one’s self-efficacy in complying with the containment measures to contrast the COVID-19 spread. In other words, the people who were inclined to evaluate themselves, their life, and the future in a positive way, were more prone to better regulating their emotions, which in turn fostered their capability to cope with COVID-19 demands.

The findings of our study suggest that positivity contributed to optimal functioning by sustaining emotional self-efficacy beliefs, and ultimately acting as a protective factor when individuals face challenges and stressful situations such as the COVID-19 pandemic. Moreover, these results are in accordance with McAdams’ [61] conceptualization of the architecture of personality, showing that self-efficacy beliefs operate at an intermediate level between broad disposition, such as positivity, and specific behavioral tendencies. In other words, an individual’s belief in their capacity to efficaciously manage their emotions contributes to transforming their positive orientation into optimal functioning, in terms of reducing the likelihood to manifest anxiety and depressive symptoms during the COVID-19 pandemic, and changing their lifestyle by adopting the containment measures to reduce the risk of illness.

### 4.4. The Moderation Effect of Age

Regarding the moderation effect of gender, we found that the posited relations among the variables did not show any significant difference between ages. In other words, no significant age effects were found in the posited pathways. This means that the effects of positivity and regulatory emotional self-efficacy on individual’s functioning were similar among young, middle, and older adults.

### 4.5. The Effects of the Gender and SES

Finally, regarding the effects of control variables, consistent with previous studies, we found that, across age, females were more vulnerable to anxiety and depression symptoms than males [8,62,63,64]. Furthermore, males perceived a stronger capability in managing their negative emotions than females [22].

In contrast, as to beliefs concerning one’s capability to express positive emotions and in complying with the containment measures, the effect of gender varied with age. In particular, females perceived a stronger capability in expressing their positive emotions in early adulthood [99], and perceived a stronger capability to cope with COVID-19 demands in middle adulthood, than men.

Regarding the effect of SES, we found that high SES is significantly associated with high positivity across age, and positively related to high capability to express positive affect in late adulthood. Results suggest that individuals who live in privileged circumstances are more prone to evaluate themselves, their life, and their future in a positive way and perceive themselves as more able to express positive emotions in response to success or pleasant events.

## 5. Conclusions

Overall, our findings, although exploratory, suggest that people who positively evaluated themselves, the past, and the future, and perceived themselves as efficacious to regulate their negative affect and express positive affect displayed a good adaption during the COVID-19 pandemic. However, the exploratory and cross-sectional nature of the study discourages conjectures about the direction of path and primacy of influence between positivity and self-efficacy beliefs. Likely, they operate in concert and reinforce each other in moderating the negative impact of the pandemic on individuals’ moods, habits, and relations. In fact, positive orientation is likely a disposition that since the beginning prepares people to deal with life under a positive stance and as such that predisposes them to experiences from which regulatory emotional self-efficacy beliefs derive. However, it is also intuitive that positive orientation can be strengthened by regulatory emotional self-efficacy beliefs as self-reflection and learning from experience allow people to change themselves if needed to give a better course to their life. Thus, both positivity and self-efficacy beliefs are malleable to change, whereas positivity may support self-efficacy beliefs, mastery experiences dictate the ways in which people can capitalize upon experience and nurture confidence in themselves, their futures, and their lives [27]. Moreover, prior studies have shown that positive orientation, despite its main trait-like nature [26], is partially malleable since it is influenced by both individual (i.e., self-efficacy beliefs) [27,28,57] and contextual factors (i.e., positive school climate) [43]. This is particularly needed in times of uncertainty and fear such as the period we are currently living in.

## 6. Limitations and Future Research

Although the present study confirmed the hypothesized relations among the variables of interest, some limitations should be acknowledged. Firstly, the reliance on only self-reported data may be viewed as a major limitation of the study. Although one may claim that no one can report about one’s own personality, self-efficacy beliefs, and subjective well-being better than that person, further research should corroborate the posited model using a multi-informant approach to minimize the possibility of inflated effects due to the same-informant bias. Another limitation is concerned with the experimental design. The use of a survey online limited the sample to only those who had internet access, leaving the population not using networked devices unexplored. Moreover, this experimental design did not register the refusal rate or estimate how many persons were reached. However, despite these limitations, the use of online recruitment was our only practical feasible sampling method during the COVID-19 lockdown, which helped us to collect data even if we were confined to our home. Another limitation is the generalizability of the present findings from the Italian population to other cultural contexts. Although all people around the world may face common demands and challenges due to COVID-19 and consequently share similar adjustment problems, further research should investigate the posited model of influences in different cultural contexts. Finally, as mentioned above, this study used a cross-sectional design and therefore the findings obtained did not allow to hypothesize the exact causal relations between the variables.

Despite the limitations, the present study has some practical implications in highlighting the relevance of improving individuals’ positive orientation and regulatory emotional self-efficacy beliefs as key factors aimed at preventing people’s adjustment problems during the COVID-19 pandemic. Specifically, the results of the present study might provide mental health and social care professionals with relevant information on protective mechanisms when dealing with the very high levels of psychological distress during the COVID-19 crisis. It is important for clinicians to be aware of the meaning-making process individuals make especially when significant distress is due to adversity or trauma. According to the Bandura’s agency framework [18], individuals are not a passive product of the environmental processes around them; on the contrary, they are the main actors in this process. This means that through agentic action, human beings find a way to flexibly adjust to the conditioned and socially diverse environments. This meaning-making process is prompted and facilitated by personality features such as positivity and self-efficacy beliefs. When individuals seek professional help during crises, clinicians might help them reframe their circumstances in a way that guides adaptive behavior through their beliefs to be able to regulate their emotions and behaviors. According to the most recent guidelines about COVID-19 health services’ organization, mental health care should be organized following principles of holism, availability, and accessibility to professional help [100,101]. Following these principles, the preliminary results from the present study might provide professionals with information on personality characteristics and processes that can be promoted to counteract the negative effects of the pandemic on people’s psychological well-being.

## Figures and Tables

**Table 1:** Means (M) and standard deviations (SD) of the study variables.

Variables	Total Sample		Early Adulthood		Middle Adulthood		Late Adulthood	
	M	SD	M	SD	M	SD	M	SD
Positivity	3.72	0.67	3.64	0.71	3.78	0.62	3.79	0.61
SE_NEG	2.94	0.70	2.89	0.68	2.99	0.75	2.97	0.68
SE_POS	3.71	0.83	3.67	0.87	3.78	0.80	3.68	0.77
State anxiety	2.07	0.57	2.15	0.61	1.99	0.53	1.98	0.49
Depressive symptoms	1.72	0.58	1.85	0.60	1.61	0.53	1.58	0.51
SE_COVID-19	4.07	0.61	3.99	0.61	4.11	0.61	4.24	0.55

Notes: SE_NEG, self-efficacy beliefs in managing negative emotions; SE_POS, self-efficacy beliefs in expressing positive emotions; and SE_COVID-19, self-efficacy in complying with the COVID-19-related containment measures.

**Table 2:** Correlations among study variables.

	1	2	3	4	5	6
1. Positivity	—					
2. SE_NEG	0.41 ***	—				
3. SE_POS	0.46 ***	0.36 ***	—			
4. State Anxiety	−0.52 ***	−0.40 ***	−0.042 ***	—		
5. Depressive symptoms	−0.52 ***	−0.38 ***	−0.36 ***	0.70 ***	—	
6. SE_COVID-19	0.17 ***	0.18 ***	0.27 ***	−0.15 ***	−0.13 ***	—

Notes: SE_NEG, self-efficacy beliefs in managing negative emotions; SE_POS, self-efficacy beliefs in expressing positive emotions; and SE_COVID-19, self-efficacy in complying with the COVID-19-related containment measures. *** *p* < 0.001.

**Table 3:** Standardized effects of gender and SES on study variables.

	Positivity	SE_NEG	SE_POS	State Anxiety	Depressive Symptoms	SE_COVID-19
Gender	−0.02; −0.06; −0.05	−0.13 ***; −0.11 *; −0.13 *	0.07 *; 0.03; −0.03	0.16 ***; 0.16 ***; 0.10 *	0.14 ***; 0.14 **; 0.22 ***	0.05; 0.12 *; 0.06
SES	0.09 *; 0.10 *; 0.12 *	0.01; 0.03; −0.05	−0.03; 0.07; 0.11 *	0.02; −0.04; −0.04	−0.02; −0.02; −0.05	0.01; 0.03; −0.06

Notes: The standardized coefficients are reported for early adulthood, middle adulthood, and late adulthood, respectively. To maintain model parsimony, only significant effects were retained in the final model. SES, socioeconomic status; SE_NEG, self-efficacy beliefs in managing negative emotions; SE_POS, self-efficacy beliefs in expressing positive emotions; and SE_COVID-19, self-efficacy in complying to the COVID-19-related containment measures. Gender: 0 (male), 1 (female). * *p* < 0.05; ** *p* < 0.01; *** *p* < 0.001.

## Data Availability

Data available on request due to restrictions, e.g., privacy or ethical. The data presented in this study are available on request from the corresponding author. The data are not publicly available due to the privacy and professional specificity of the people who took part in this research.
